# Evaluation of Liver Ischemia-Reperfusion Injury in Rabbits Using a Nanoscale Ultrasound Contrast Agent Targeting ICAM-1

**DOI:** 10.1371/journal.pone.0153805

**Published:** 2016-04-27

**Authors:** Fang Xie, Zhi-Ping Li, Hong-Wei Wang, Xiang Fei, Zi-Yu Jiao, Wen-Bo Tang, Jie Tang, Yu-Kun Luo

**Affiliations:** 1 Department of Ultrasound, Chinese PLA General Hospital, Beijing, China; 2 Department of Ultrasound, North China University of Science and Technology Affiliated Hospital, Tangshan, China; 3 Pharmacology Research Department, Beijing Institute of Pharmacology and Toxicology, Beijing, China; IDIBAPS - Hospital Clinic de Barcelona, SPAIN

## Abstract

**Objective:**

To assess the feasibility of ultrasound molecular imaging in the early diagnosis of liver ischemia-reperfusion injury (IRI) using a nanoscale contrast agent targeting anti-intracellular adhesion molecule-1 (anti-ICAM-1).

**Methods:**

The targeted nanobubbles containing anti-ICAM-1 antibody were prepared using the avidin-biotin binding method. Human hepatic sinusoidal endothelial cells (HHSECs) were cultured at the circumstances of hypoxia/reoxygenation (H/R) and low temperature. The rabbit liver IRI model (I/R group) was established using the Pringle’s maneuver. The time-intensity curve of the liver contrast ultrasonographic images was plotted and the peak intensity, time to peak, and time of duration were calculated.

**Results:**

The size of the targeted nanobubbles were 148.15 ± 39.75 nm and the concentration was 3.6–7.4 × 10^9^/ml, and bound well with the H/R HHSECs. Animal contrast enhanced ultrasound images showed that the peak intensity and time of duration of the targeted nanobubbles were significantly higher than that of common nanobubbles in the I/R group, and the peak intensity and time of duration of the targeted nanobubbles in the I/R group were also significantly higher than that in the SO group.

**Conclusion:**

The targeted nanobubbles have small particle size, stable characteristic, and good targeting ability, which can assess hepatic ischemia-reperfusion injury specifically, noninvasively, and quantitatively at the molecular level.

## Introduction

Ischemia-reperfusion injury (IRI) is an important postoperative complication of liver transplantation and resection, and greatly compromises the graft survival and postoperative liver function. IRI includes hypoxia-induced cell damage and inflammatory immune factor-induced progressive cell damage after reoxygenation. Currently, studies on the hepatocyte injury and its abnormal regulation have been reported [1]. Subsequently, the damage of nonparenchymal cells, especially liver sinusoidal endothelial cells, was gradually calling people’s attention [2].

Liver sinusoidal endothelial cells (LSECs) located in the sinusoidal blood vessel wall play an important role in regulating the material transfer of hepatic microcirculation, inflammatory reactions, and the removal of metabolic wastes. Their unique anatomical location and physiological characteristics constitute the pathophysiological basis of liver IRI [3, 4]. In the early stage of liver IRI, the LSECs are damaged prior to the parenchymal cells [5].

It has been shown that LSECs were more sensitive to IRI in the cold preservation process of liver transplantation [6–8]. Liver IRI is essentially a series of inflammatory reactions. Intracellular adhesion molecule-1 (ICAM-1) is a membrane protein of the immunoglobulin superfamily [9]. ICAM-1 is expressed in the sinusoidal endothelial cells [10] and is involved in signal transduction, cell adhesion, inflammation, thrombosis, and wound healing [11]. ICAM-1 is normally expressed at a low level in liver, but is upregulated in the absence of oxygen [12].

Although the molecular mechanisms of IRI have been studied thoroughly [13], there is still a lack of early specific diagnosis indicators. Imaging of the intrahepatic small blood vessels using routine ultrasound or Doppler ultrasound is sometimes dissatisfactory. In recent years, contrast-enhanced ultrasonography (CEUS) has dramatically improved the imaging of small blood vessels [14–18]. The microbubble-based contrast agents currently used in clinical practice lack affinity for the lesions, resulting in imaging duration only about 2–5 min [19–21].

With the development of targeted ultrasound contrast agents and the appearance of nanobubbles, the ultrasound molecular imaging technique has undergone a revolutionary progress and become the focus of ultrasound applications [22]. Its main principle is to intravenously inject targeted nanobubble contrast agent carrying specific ligands, which is selectively accumulated in the lesions.

The present study aimed to prepare a nanobubble ultrasound contrast agent targeting ICAM-1, and examine its binding ability to HHSECs *in vitro* and its imaging ability *in vivo*.

## Materials and Methods

### Preparation of the nanobubbles

The targeted nanobubbles were prepared using the thin-film hydration-sonication method [23]. Briefly, HSPC, DSPE and Bio-DSPE-PEG 2000 (85:5:10, W/W/W; Advanced Vehicle Technology Pharmaceutical, Shanghai, China) were dissolved in 1 ml chloroform. The solution was evaporated in vacuum to form a thin film. The film was hydrated with 1 ml phosphate-buffered saline (PBS), dried again in vacuum overnight, and maintained at 55°C in a shaking incubator for 45 min to form liposomes. The liposomal suspension was transferred into a 1.5-ml tube, and the air above the liquid was replaced with C_3_F_8_ gas using a 5-ml syringe equipped with a long and fine needle. Finally, the solution was sonicated at 95 W for 8 s. The biotinylated lipid nanobubbles were obtained after the large bubbles were separated as a thin layer from the suspension by low-speed centrifugation. According to the biotin-avidin bridging chemistry method [24, 25], streptavidin was added into the biotinylated lipid nanobubble suspension at a ratio of 1:1 (mol/mol) and incubated at 4°C for 30 min. Tabbit biotinylated anti-ICAM-1 antibody (Biosynthesis Biotechnology, Beijing, China) was added into the solution at a ratio of 1:20 (W/W) and a final concentration of 10 μg/ml. The targeted nanoscale ultrasound contrast agent was acquired after incubation at 4°C for 40 min.

The common nanoscale ultrasound contrast agent was prepared with the thin-film hydration-sonication method as described above, except that DSPE-PEG 2000 was used instead of Bio-DSPE-PEG 2000.

The targeted nanobubbles (100 μl) containing anti-ICAM-1 antibody and the common nanobubbles without anti-ICAM-1 antibody (100 μl) were mixed with FITC-labeled goat anti-rabbit IgG (Biosynthesis Biotechnology) at a dilution ratio of 1:100, and incubated at room temperature for 90 min in dark. It was then washed three times with PBS in dark.

### Characterization of the nanobubbles

The fluorescent distribution of the antibodies on the targeted nanobubbles were photographed under a laser scanning confocal microscope (TCS SP5, Leica, Germany), with common nanobubbles not labeled with antibodies as the control group. The nanobubbles were examined using a transmission electron microscope (Hitachi H-7650, Tokyo, Japan) and a laser particle size analyzer (Zetasizer Nano ZS, Malvern Instruments, Worcestershire, UK). The nanobubbles concentration was measured using an Archimedes particle measurement and analysis system (Malvern Instruments, Worcestershire, UK).

### Cell culture

HHSECs (ScienCell Research Laboratories, San Diego, CA, USA; Catalog Number 5000, Lot Number 13393) frozen in the liquid nitrogen were rapidly put into a 37°C water bath for recovery. The cell suspension was transferred into a flask coated with human fibronectin (ScienCell Research Laboratories) to a density of 5000 cells per cm^2^. An endothelial cell medium (ScienCell Research Laboratories) containing 5% fetal bovine serum was used. The cells were cultured at 37°C with 5% CO_2_ until 90% confluence. Then the cells were digested, centrifuged, resuspended, and inoculated in 40 35-mm dishes. The cells were allowed to grow until 50% confluence.

### Modeling of cool hypoxia/reoxygenation injury

The 40 petri dishes were randomly divided into the H/R group (*n* = 20) and the control group (*n* = 20). The serum-contained media of the H/R group were replaced with serum-free, sugar-free DMEM media (Gibco, Waltham, MA, USA). The pH was adjusted to 6.2. The H/R group suspensions were transferred into a closed anaerobic culture tank (Mitsubishi, Tokyo, Japan), placed in a 4°C refrigerator with an O_2_ concentration of < 0.1%, reoxygenated 24 h later, replaced to serum-contained endothelial cell medium (pH 7.4), and then transferred to a 37°C, 5% CO_2_ incubator to culture for 4 h. Cells in the control group were cultured in 37°C, 5% CO_2_ incubator for 28 h.

### Cell immunofluorescence

The HHSECs were fixed with 4% paraformaldehyde. Unlabeled anti-ICAM-1 antibody was added to each group overnight at 4°C. FITC-labeled goat anti-rabbit IgG was added for 90 min at room temperature. The nuclei were counterstained using DAPI (Beyotime Institute of Biotechnology). The immunoreactivity of ICAM-1 protein was photographed under a laser confocal microscope.

### In vitro binding test

After the cells were fixed, each group was randomly added with 100 μl of prepared targeted nanobubbles or unlabeled common nanobubbles (four groups), incubated at room temperature for 60 min. FITC-labeled goat anti-rabbit IgG was added at room temperature for 90 min. The nuclei were stained with DAPI. The binding of the two nanobubbles to the HHSECs was photographed under a laser confocal microscope.

### Animal model of IRI

Twenty male or female New Zealand rabbits, weighing 3.0–3.5 kg, were provided by Chinese PLA General Hospital Experimental Animal Center (Experimental animal license number: SCXK [Beijing] 2010–0001). All the rabbits were allowed to accommodate for 2 weeks with free access to food and water under 12/12 h light/dark cycles. The study protocol was approved by the Experimental Animal Welfare Ethical Review Committee of the Chinese PLA General Hospital (2015-x10-11).

The animals were randomly divided into the experimental group (*n* = 10) or the sham-operation (SO) group (*n* = 10). All the rabbits were fasted, but had free access to water, for 12 h before operation. Then they were anesthetized by injecting 3% sodium pentobarbital at a dose of 30 mg/kg via the ear vein. For the experimental group, Pringle’s maneuver [26] was used to establish the normothermic liver IRI model by ischemia for 60 min and reperfusion for 120 min. For the SO group, laparotomy was performed without clamping the hepatic artery, portal vein, and bile duct, and the abdomens were closed 60 min later. After the vascular clamp of the rabbit in the I/R group was released to reflow for 5 min, the liver color turned from brown to red.

### Contrast-enhanced ultrasonography

Contrast-enhanced ultrasonography was performed using the common nanobubbles, and using the targeted nanobubbles 1 h later (Philips iU Elite ultrasound imaging system and L_12-5_ transducer, Philips Medical Systems, Bothel, WA, USA). Before the examination, a 1.5-ml dose of common or targeted nanobubbles was bolus injected via the ear vein through a 24-gauge needle, followed by a flush of 2 ml normal saline. A cross section of the left lobe of the liver was selected as the observation area, and the contrast side/side mode was performed. The modes of contrast-enhanced sonographic imaging were the same for both contrast agents with mechanical index (MI) 0.07 and depth of imaging 5.0 cm. The CEUS images were analyzed using the QLAB software (Philips Medical System, Bothel, WA, USA). The time-intensity curve was used to calculate the peak intensity, time to peak, and the time of enhancement duration (time of duration).

Image analysis was performed by two investigators with 10-year-experience of abdominal ultrasound diagnosis and 1-year-experience of CEUS, respectively, who were blind to the study design. Dispute was discussed with a third senior physician. Each animal was examined three times and the average was calculated.

### Histological examination

The animals were anesthetized and the abdomen was reopened. 2 ml venous blood was collected from the liver inferior vena cava. Serum levels of alanine aminotransferase (ALT) and lactate dehydrogenase (LDH) were examined using an automatic biochemical analyzer (Hitachi 7600, Tokyo, Japan). The rabbits were sacrificed by exsanguination. One piece of liver tissue of 1.5 × 1.5 × 0.5 cm^3^ was harvested, fixed with 4% paraformaldehyde, paraffin-embedded, and sectioned. Hematoxylin and eosin (HE) staining, immunohistochemical staining, and TUNEL assay were performed. Fore transmission electron microscopy, 1 mm^3^ liver tissue was quickly cut, fixed with 3% glutaraldehyde, and double-dyed with UO_2_Ac_2_ (30 min) and PbNO_3_ (10 min). For Western blotting, 50 mg liver tissue was cut, rinsed with ice-cold PBS, placed in freezing tubes, and stored at -80°C. The Gel-Pro 4.0 version gel optical analysis software was used to obtain the reference value of the integrated optical density (IOD) of the I/R and SO groups. The Image-Pro Plus 6.0 software was applied to analyze each picture to obtain the percentage of positive apoptotic cells in the hepatocytes and liver sinusoidal endothelial cells (positive apoptotic cells/total cells = apoptosis rate). The severity of liver IR was evaluated using the Suzuki’s score [27]. Each specimen was examined by two experienced pathologists blind to the study design.

### Statistical analysis

Continuous data were expressed as mean ± standard deviation. Comparisons were made using the independent sample *t* test or the analysis of variance followed by post hoc test of the Bonferroni method. All statistical analyses were performed using the SPSS 17.0 software. *P* < 0.05 was considered statistically significant.

## Results

### Physical properties of the contrast agents

**Fig 1:**
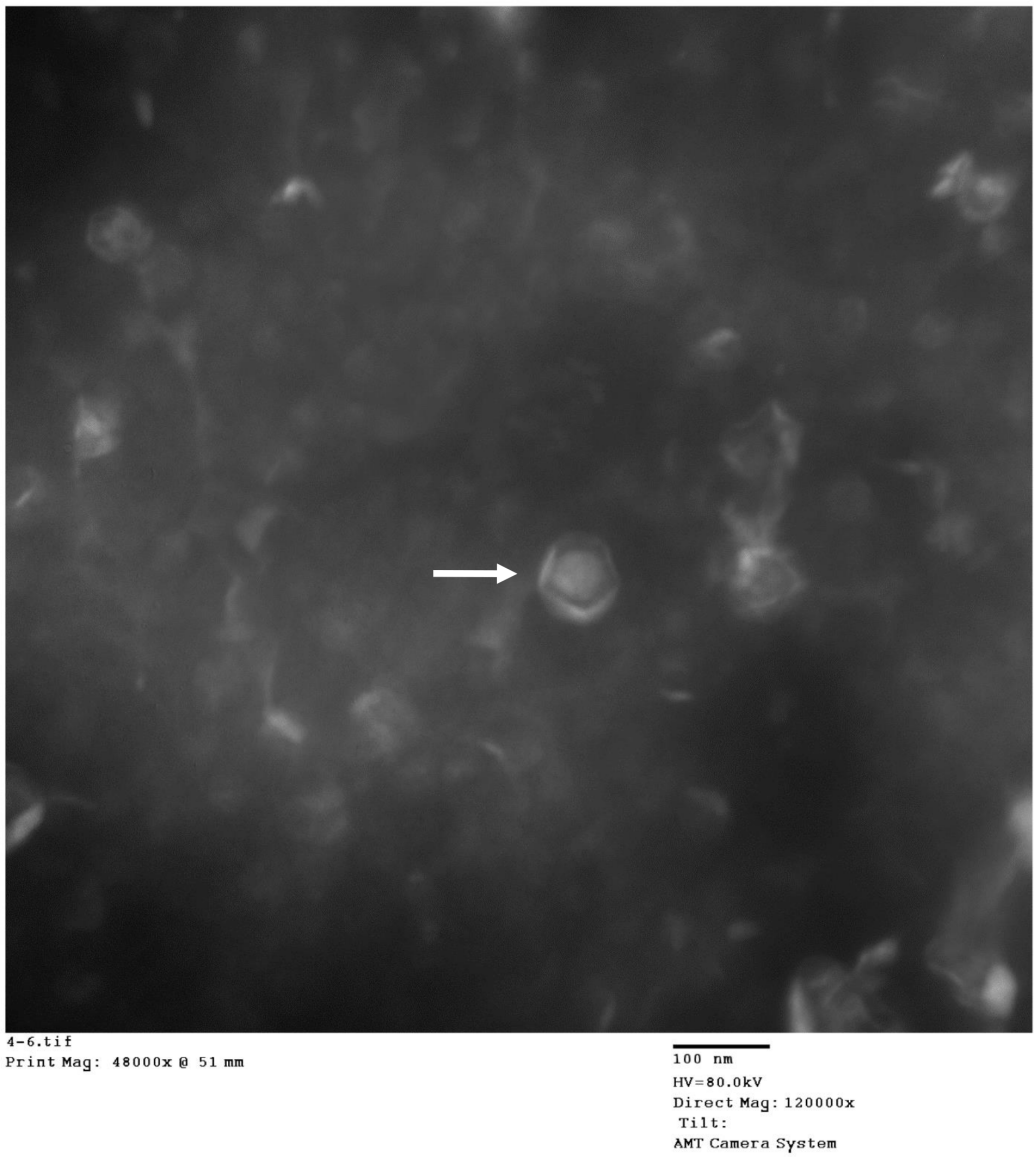
Transmission electronic microscopy shows that nanobubbles (arrow) have equal sizes and are well distributed (120, 000×).

**Fig 2:**
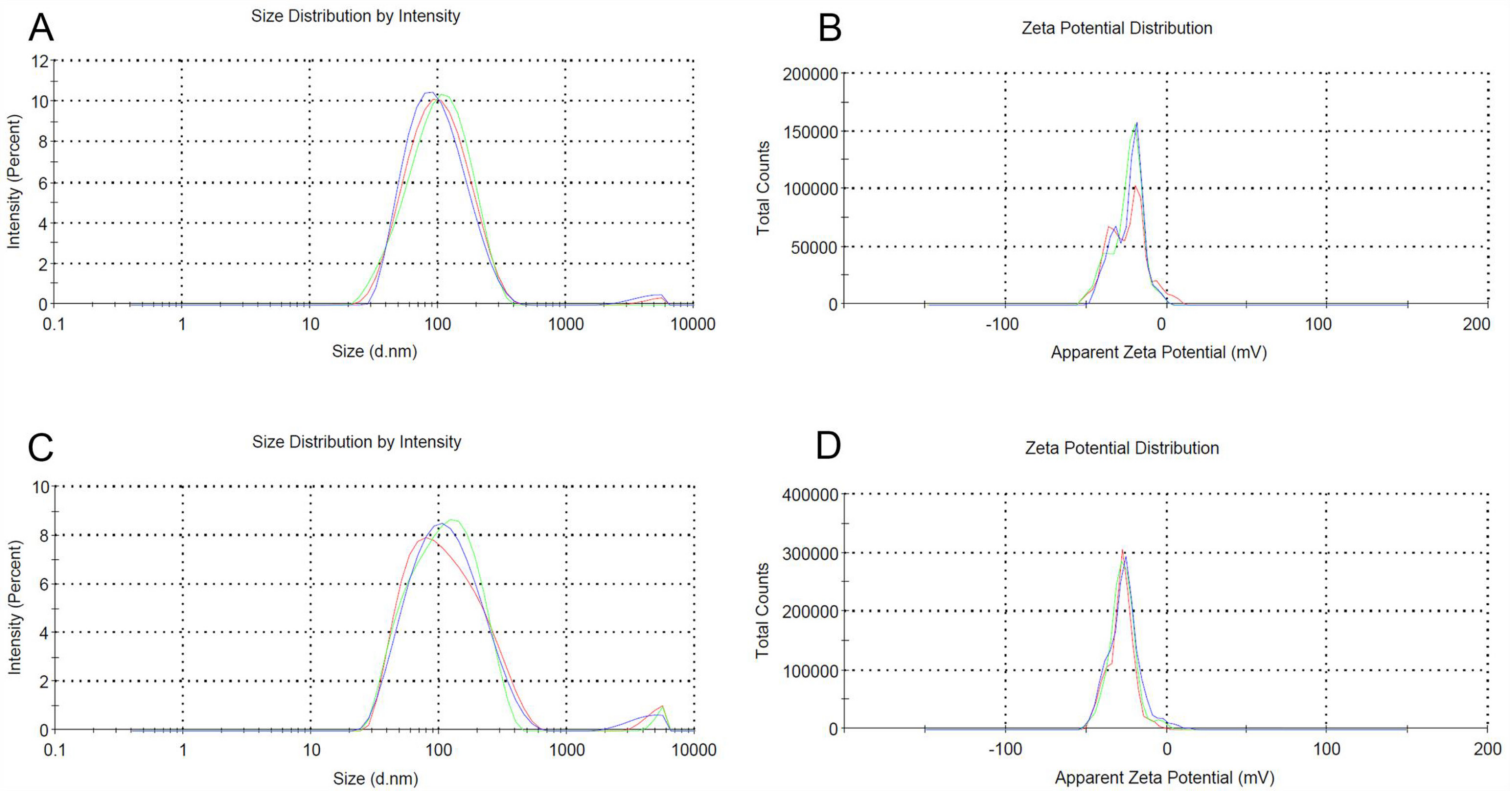
Malvern nano laser particle size analyzer detects that the particle size is 113 nm (A) and Zeta potential is -19.1 ± 4.5mV (B) for common nanobubbles, and 134 nm (C) and -28.4 ± 7.9mV (D), respectively, for targeted nanobubbles.

**Fig 3:**
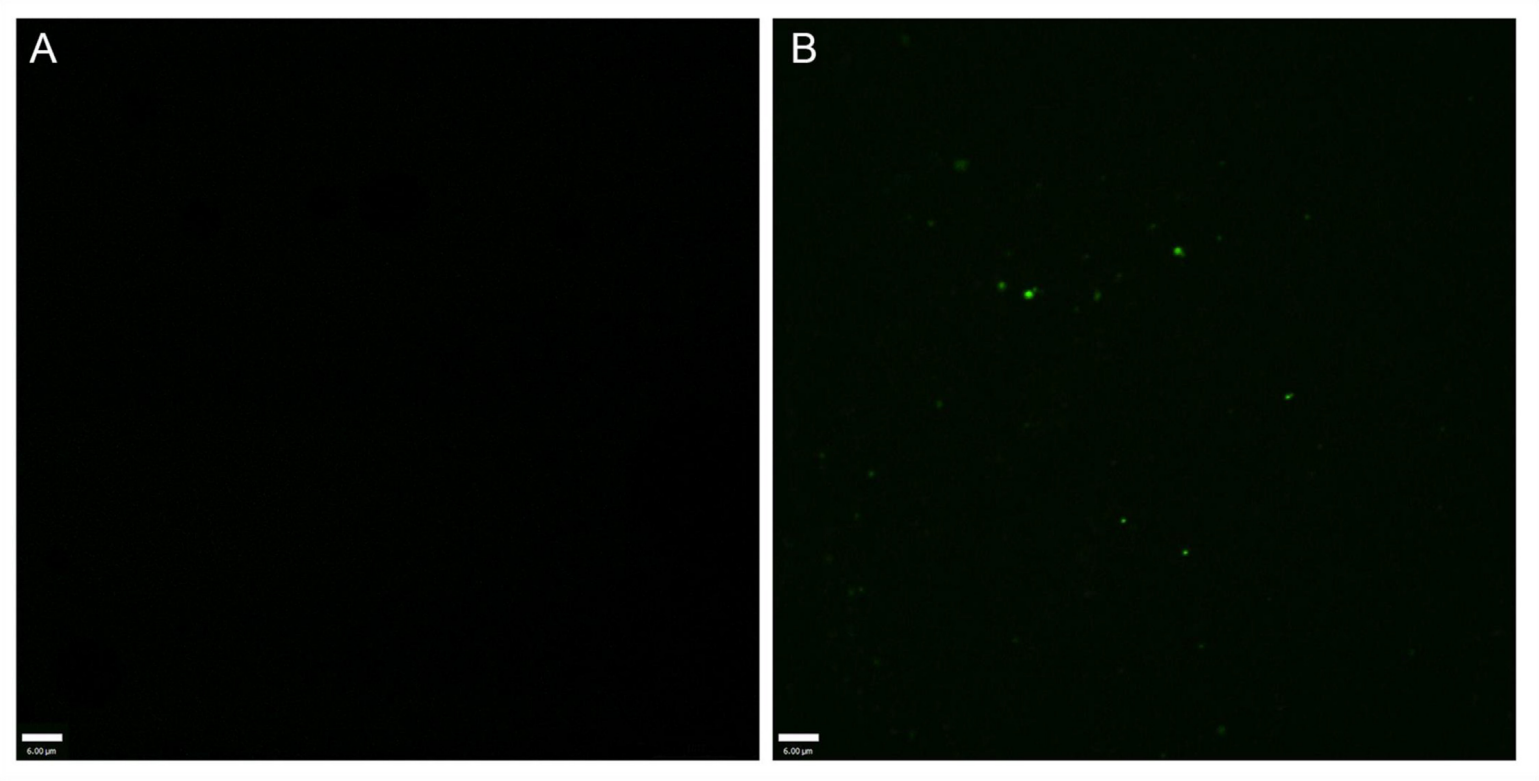
A confocal microscope shows no green fluorescence (600×) on the surface of common nanobubbles (A), but does on the surface of targeted nanobubbles (600 ×) (B).

The suspension of the contrast agents was white and opaque. The nanobubbles were evenly distributed and sized (Fig 1). The particle size of the nanobubbles was 148.15 ± 39.75 nm for the targeted nanobubbles and 129.65 ± 24.52 nm for the common nanobubbles, respectively. The Zeta potential on the surface of the nanobubbles was -28.4 ± 7.9 mV for the targeted nanobubbles and -19.1 ± 4.5 mV for the common nanobubbles, respectively (Fig 2). The concentration of nanobubbles was 3.6–7.4 × 10^9^ bubbles/ml using. No green fluorescence was observed on the surface of common nanobubbles under the confocal microscope, but was observed on the surface of the targeted nanobubbles (Fig 3).

### H/R injury characteristic of HHSECs

**Fig 4:**
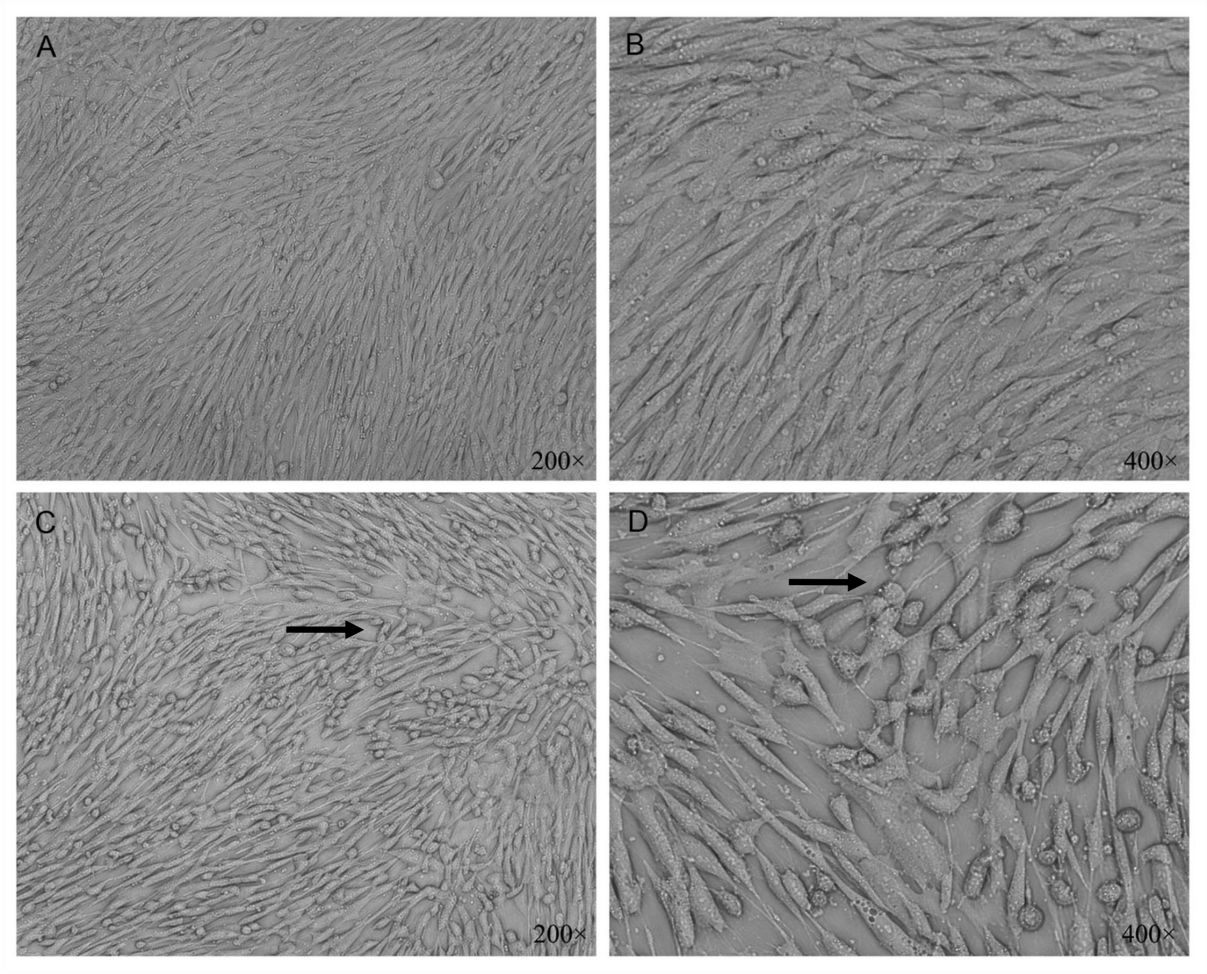
An inverted phase microscope shows that HHSECs in the control group are spindle shaped and arranged like “cobblestone” (200×) (A). The cells develop monolayer growth and the nucleus is placed in the center (400×) (B). Most of HHSECs in the H/R group retract to round shape (arrow), and the gaps of cells become widened (200×) (C). Intracellular particles increase, and some cells are dropped off (arrow) (400×) (D).

The HHSECs in the control group were fusiform or spindle shaped. The cells were arranged like “cobblestone,” grew in monolayer, and had nuclear protrusion in the center. Most HHSECs in the H/R group showed characteristic of cell death or apoptosis (Fig 4).

### Immunofluorescence test

**Fig 5:**
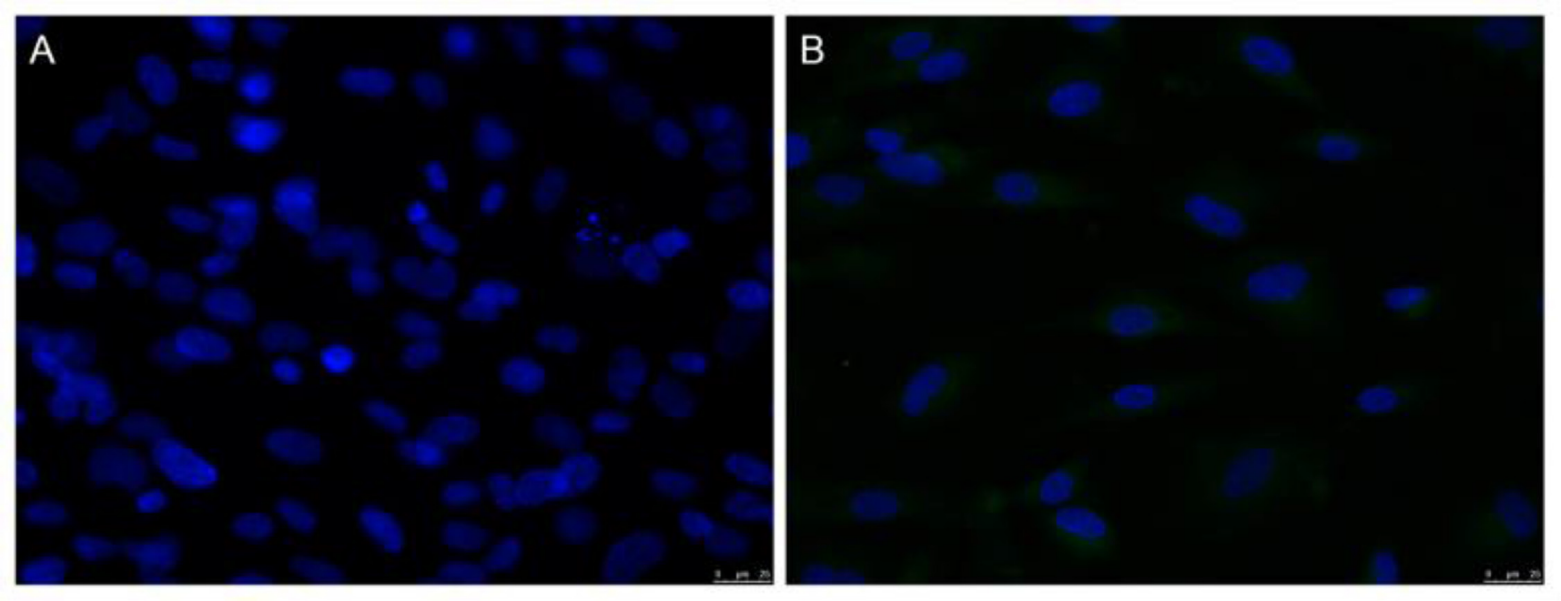
It is observed under laser confocal microscope that green fluorescence (600×) is not seen around HHSECs in the control group (A), but in the membranes and the cytoplasm of HHSECs in the H/R group (B).

Green fluorescence was not observed in the HHSECs of the control group, but was seen in the cell membrane and cytoplasm of HHSECs in the H/R group (Fig 5).

### Binding of the targeted nanobubbles and the cells

**Fig 6:**
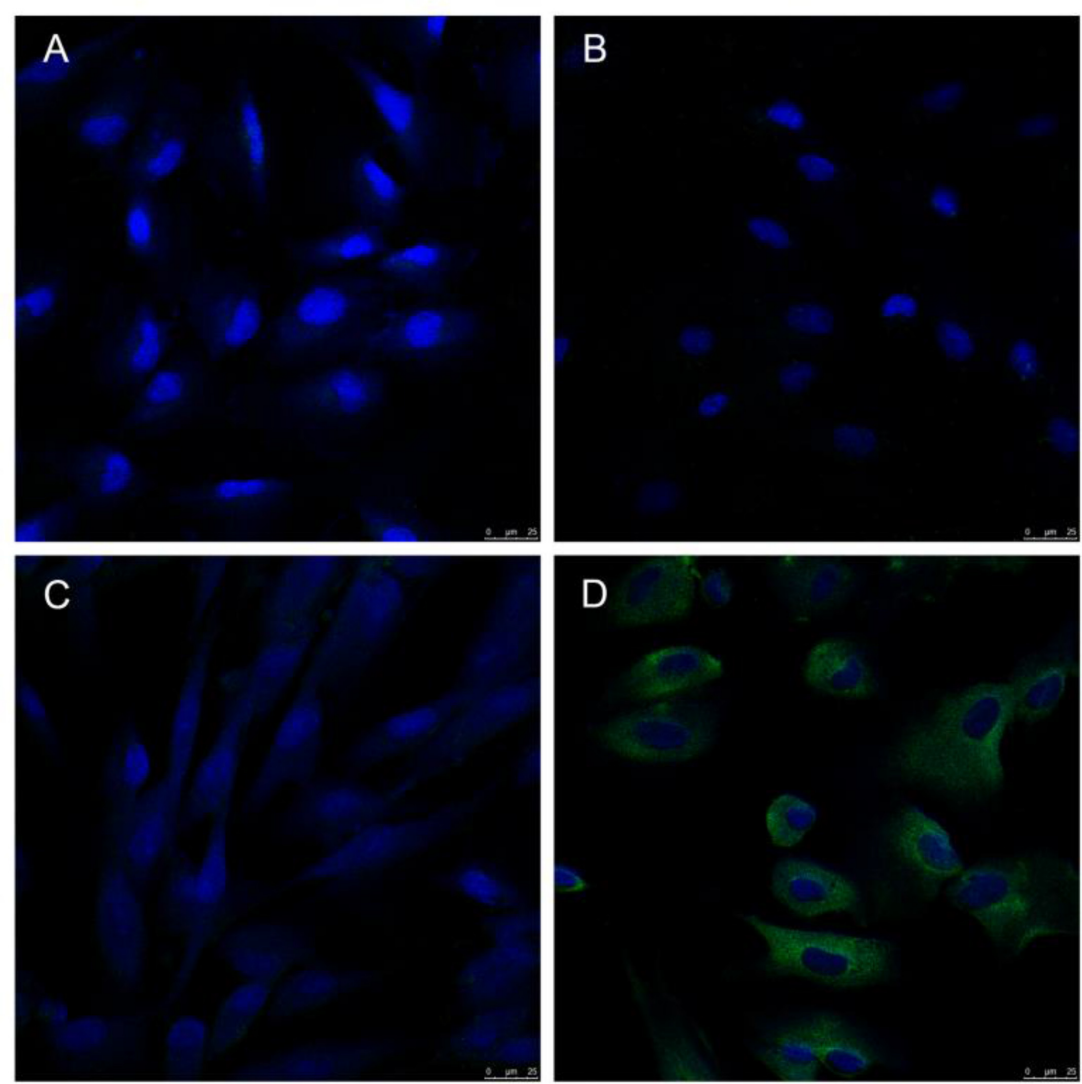
It is observed under confocal microscopy that no binding of green fluorescence and cell is observed between common nanobubbles and cells of the control group, between common nanobubbles and cells of the H/R group, or between targeted nanobubbles and cells of the control group (600×) (A, B, C). The green fluorescence from targeted nanobubbles is adhered to the cell membrane surface and intracytoplasma of HHSECs in the H/R group (600×) (D).

The targeted nanobubbles containing anti-ICAM-1 antibody adhered well to the surface and cytoplasm of HHSECs in the H/R group. However, binding was hardly observed between the common nanobubbles and control cells or the H/R cells, or between the targeted nanobubbles and the control cells (Fig 6).

### Macroscopic observation of the liver

**Fig 7:**
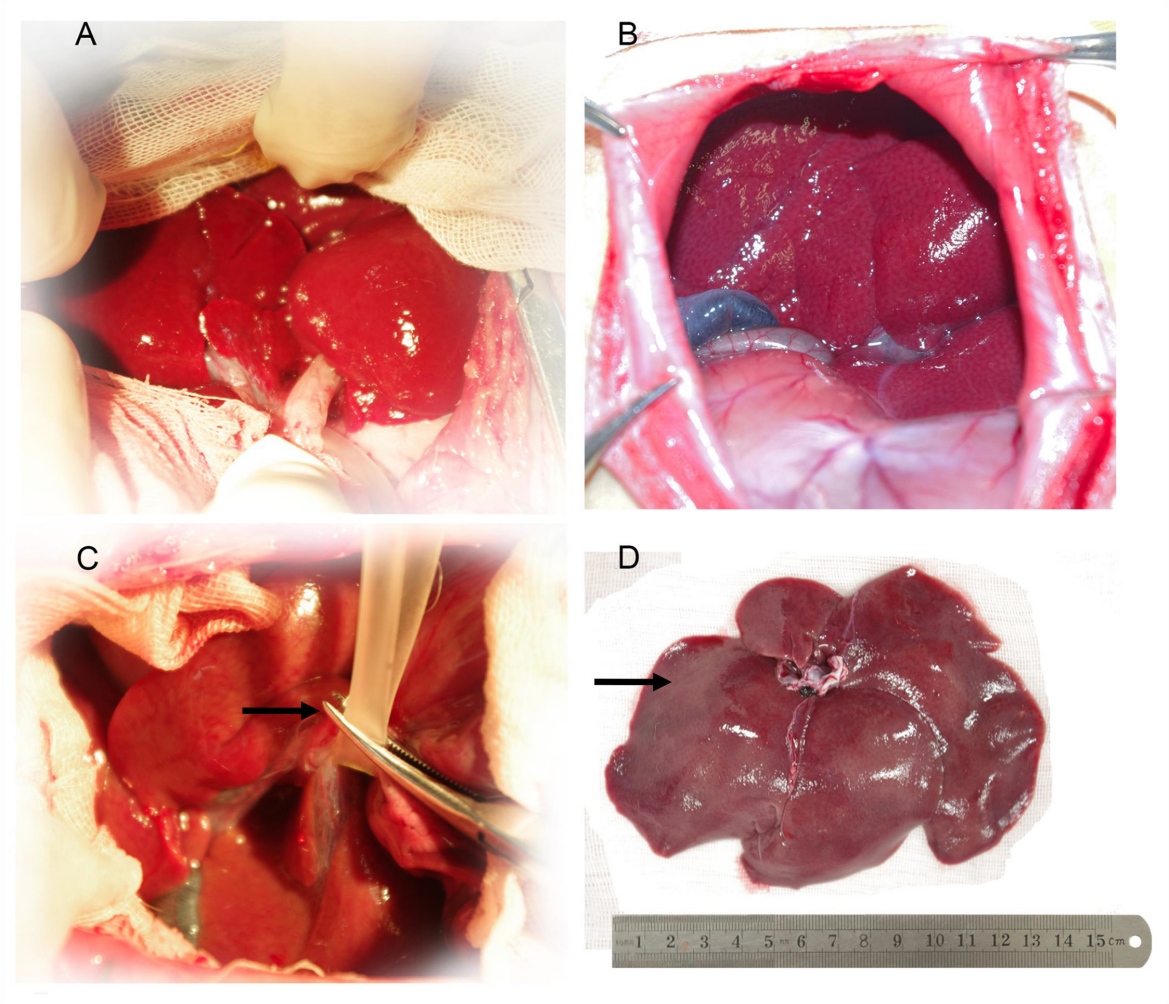
The liver is red before operation (A). The liver is normal in the SO group when abdomen is closed at 60minutes (B). The hepatic artery, portal vein, and bile duct in the I/R group are blocked (arrow) (C). The liver is swollen in the I/R group,120minutes after reflowing; the edge is blunt and the color turns from red to brown; small spotty or patchy necrosis (arrow) are scattered on the cell surface, mainly accumulating on the edge of the liver and the liver porta (D).

The livers of the SO group showed a normal morphology. The livers in the I/R group were ruddy before being blocked and were swollen after reflowing. The edges turned blunt, and the colors turned from red to brown. Small spotty or patchy necrosis were scattered on the surface, accumulating on the edges and the liver porta (Fig 7).

### Liver function tests

**Fig 8:**
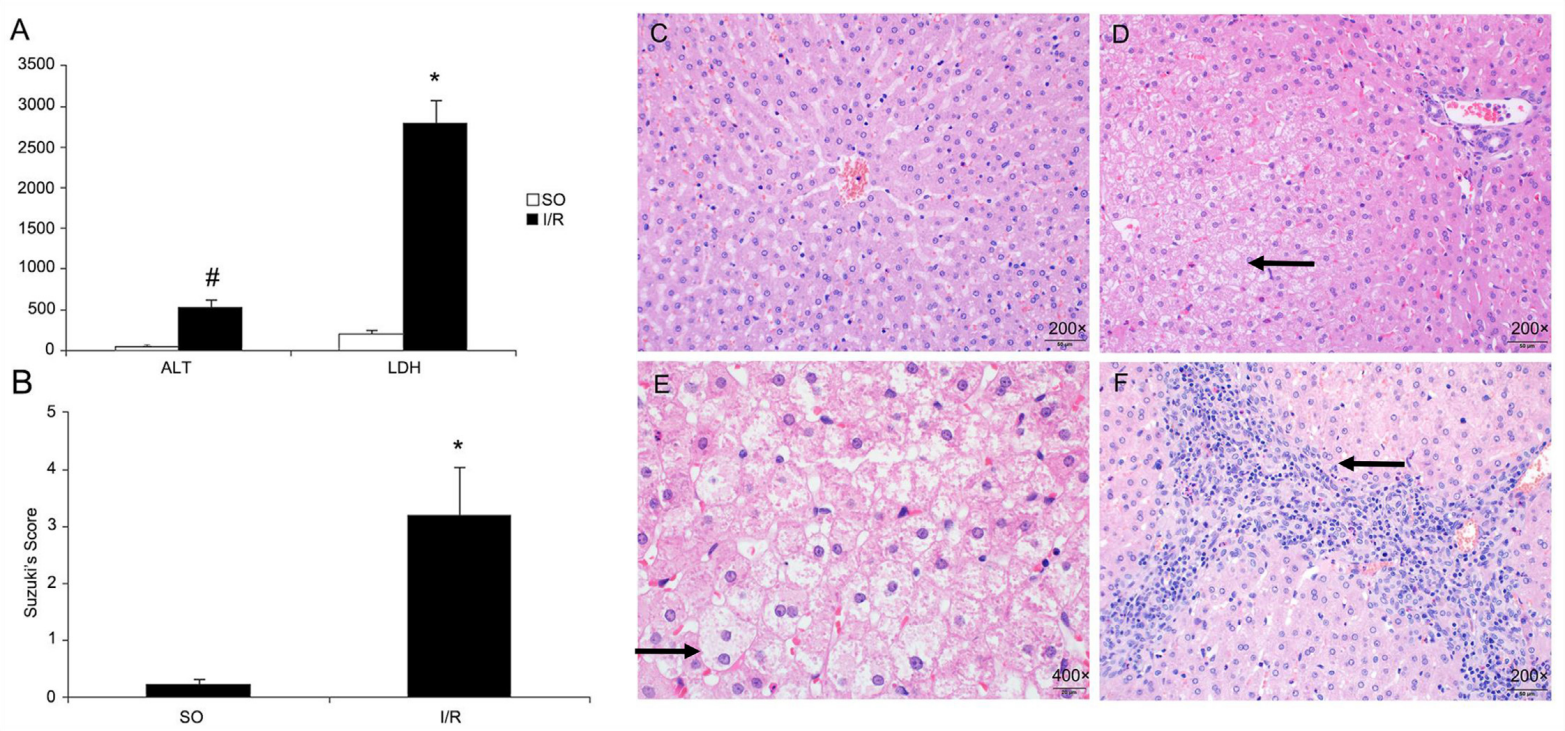
Serum ALT^**※**^ and LDH^**#**^ are significantly higher in the I/R group than in the SO group (A). The Suzuki’s score of the liver ischemia–reperfusion injury (B). Hepatic lobes in the SO group have no significant change; the nucleus are round and placed in the middle; nuclear membrane is clear; and the cytoplasm is stained red (200×) (C). Hepatocytes in the I/R group have edema; the volume becomes large(arrow); hepatic cord is transected and disorganized (200×) (D). Cytoplasm becomes light colored and extensively loose; vacuolar change is seen (arrow); microvascular structures are damaged (400×) (E). Periportal inflammatory cells (arrow) are seen (200×) (F).

Compared with the SO group, serum levels of ALT and LDH significantly increased in the I/R group 2 hours after reflowing (*P* < 0.05, Fig 8).

### HE staining and Suzuki’s score

There is no significant changes in the SO group under the light microscope. The nuclei were round and in the center of the cytoplasm. The nuclear membrane was clear, and the cytoplasm was stained red. However, in the I/R group, hepatocytes were swollen (Fig 8). The severity of the pathological injury of liver IR was evaluated by the Suzuki’s score (Fig 8).

### Microstructure under transmission electronic microscope

In the SO group, the hepatocyte structure was clear and intact. The nucleoli were in the middle of the cytoplasm, and the nucleolus and nuclear membrane were clear. The cytoplasm, mitochondria, and rough endoplasmic reticulum were in order. The shape and structure were normal. The crest was clear and junctions were tight. The cholangiole microvilli were seen.

**Fig 9:**
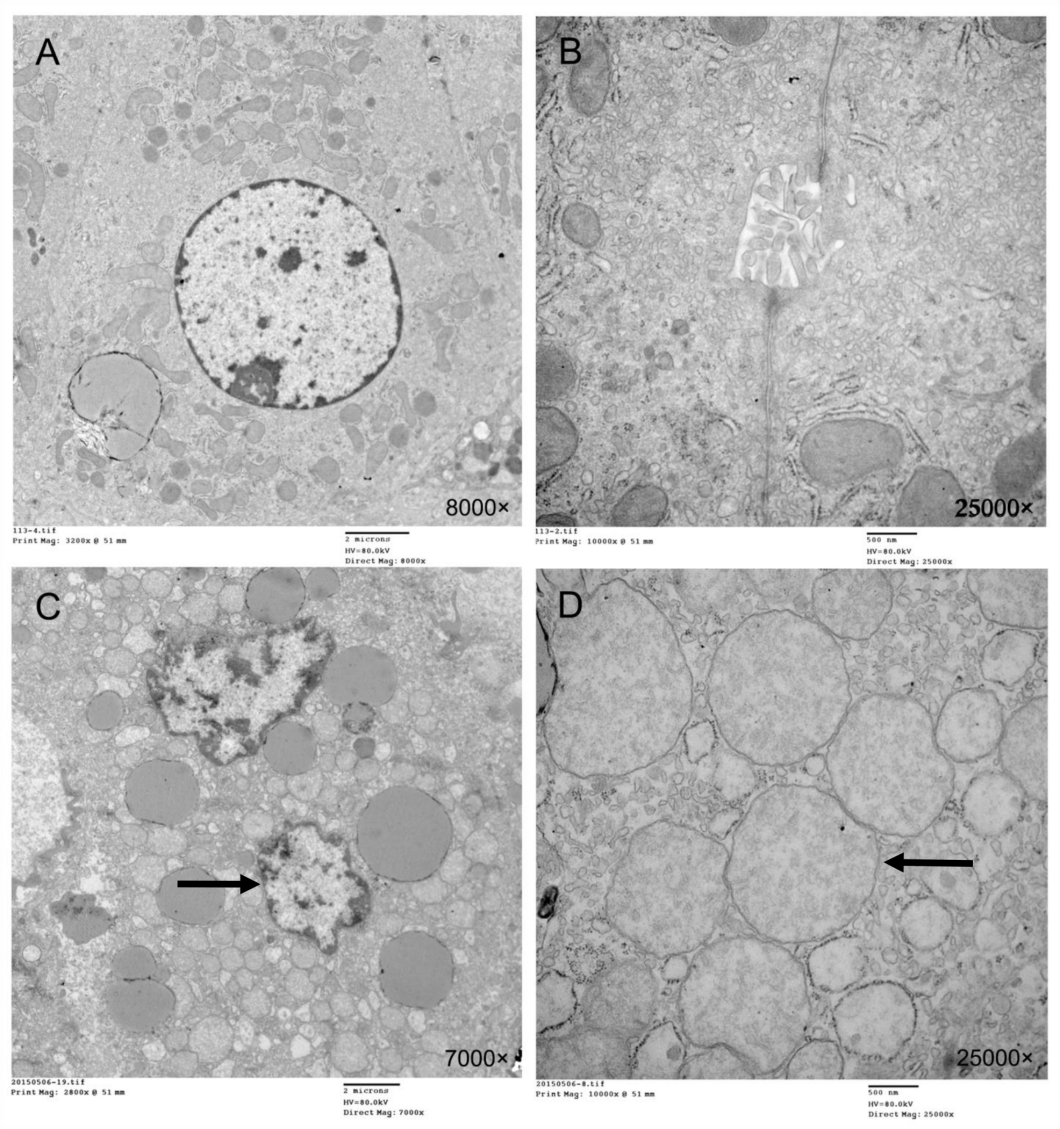
Transmission electronic microscopy reveals that the hepatocytes in the SO group have a clear and complete structure, nuclei are placed in the middle, nucleolus and nuclear membrane are clear, and cytoplasm mitochondria and rough endoplasmic reticulum are well arranged with a normal shape and structure. The ridges are clear (8000×) (A). Hepatocytes in the SO group have a tight junction and normal cholangiole microvilli can be seen (25,000×) (B). The I/R group is shown to have irregular nucleus and karyopyknosis, expanded perinuclear space, chromatin margination and condensation (arrow), lost tight junctions, and breaking and dropping off of microvilli (7000×) (C). Significantly swollen mitochondria (arrow); vacuoles; blurred, disappearing, or decreasing crest; expanded rough endoplasmic reticulum; and large particle lipid droplets (25,000×) are also seen in the IR group (D).

Mitochondria in the hepatocytes were shown to be significantly swollen under the microscope in the I/R group. Vacuoles appeared and the crest was blurred, reduced or even disappeared. Rough endoplasmic reticulum increased in size, and large particle lipid droplets were seen. Karyopyknosis appeared and the nucleus gap enlarged. The chromatin was concentrated. The tight junctions were lost, and microvilli broke and dropped off (Fig 9).

### Immunohistochemical staining

**Fig 10:**
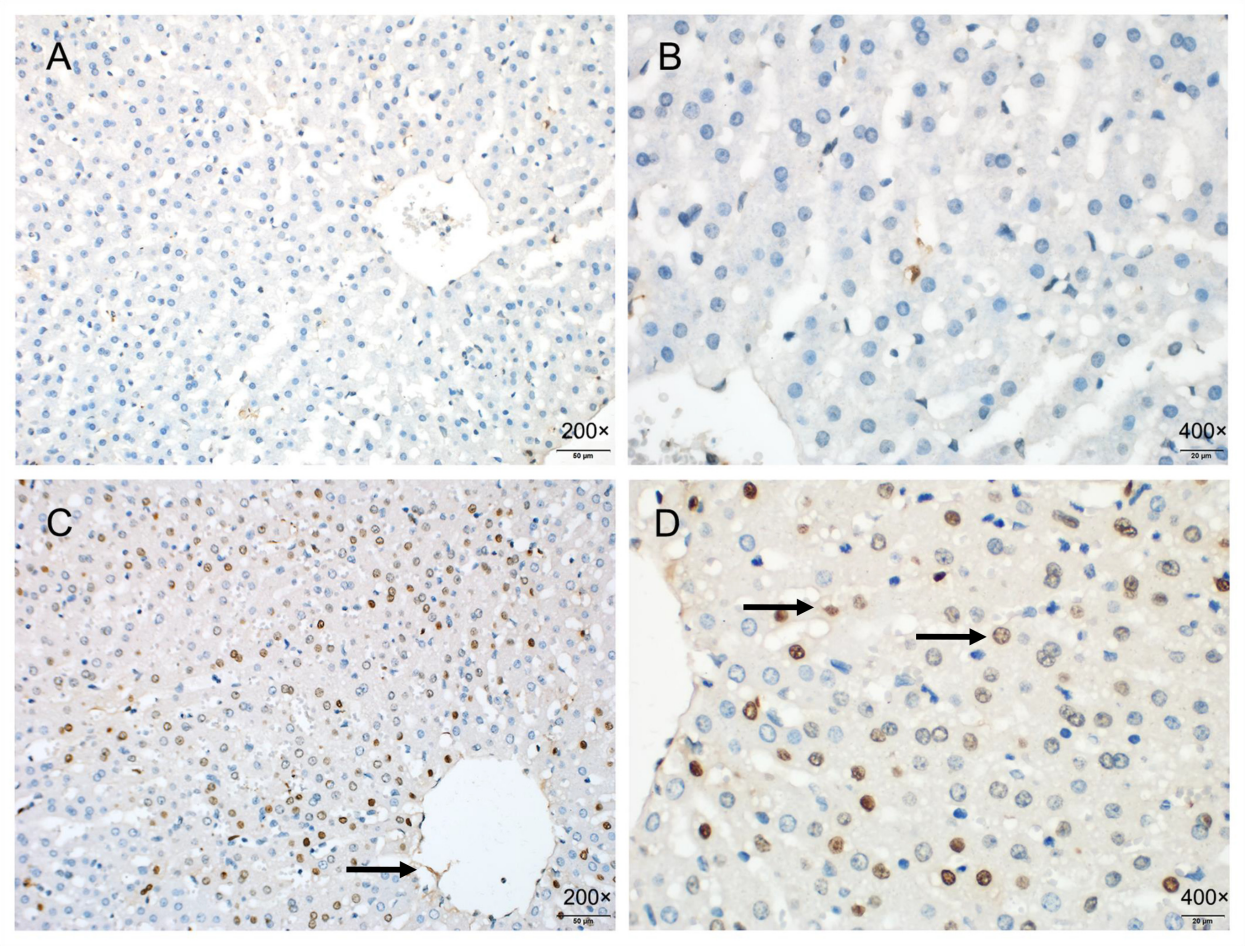
Immunohistochemistry shows that no obvious ICAM-1 staining (200×, 400×) is seen in the liver samples of the SO group (A, B); and ICAM-1 has a high expression in periportal endothelial cells (arrow), liver sinusoidal endothelial cells (arrow), and some hepatocytes (arrow) in the I/R group, with significantly deep staining and expanded area (200×, 400×) (C, D).

The staining of ICAM-1 was hardly seen in the liver samples of the SO group. However, the periportal endothelial cells, liver sinusoidal endothelial cells, and some hepatocytes in the I/R group were deeply stained (Fig 10).

### TUNEL staining

**Fig 11:**
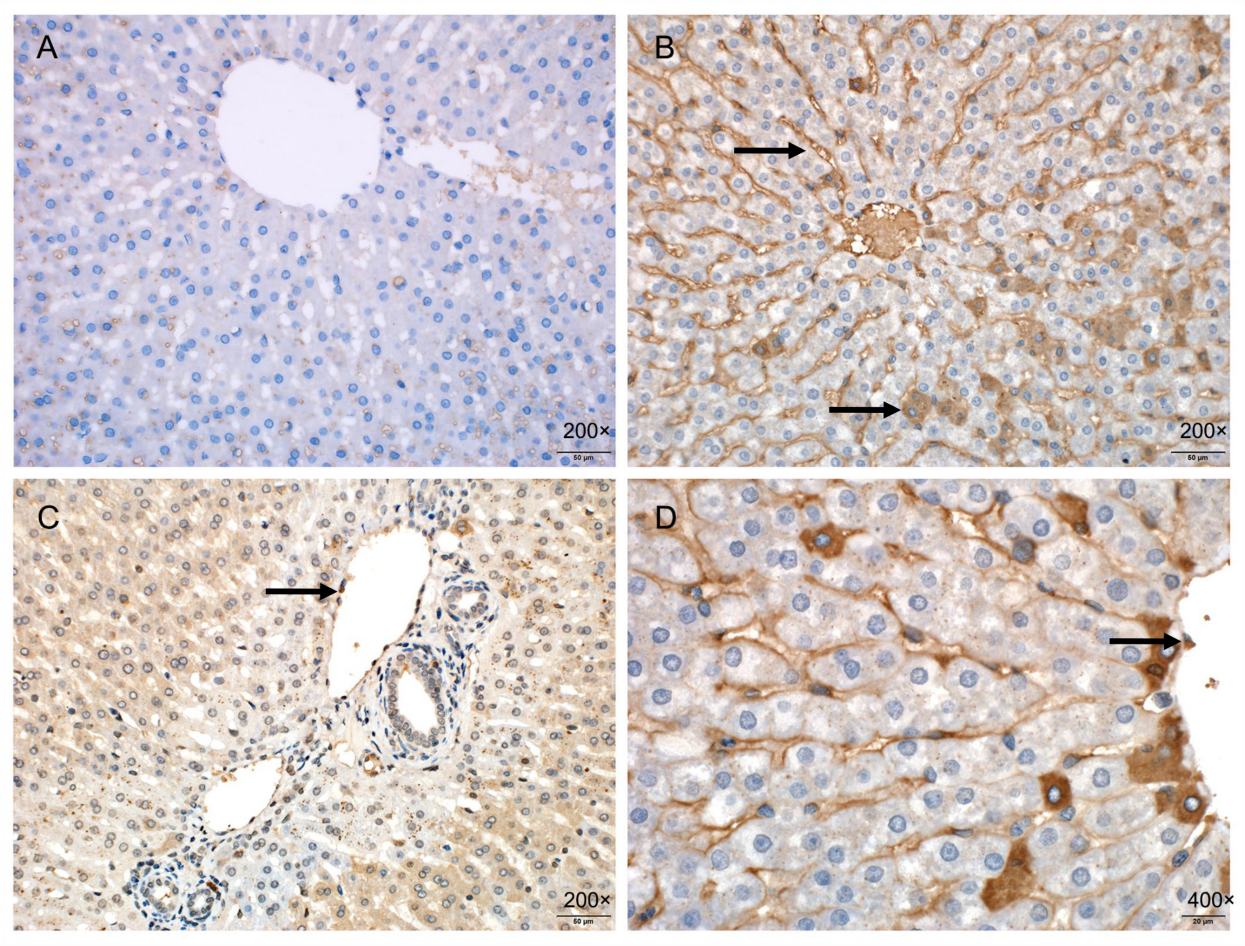
TUNEL staining shows that the level of apoptosis is lower in the SO group (200×) (A); the liver sinusoidal endothelial cells (arrow) and some hepatocytes (arrow) in the I/R group are in apoptosis; apoptotic cells have karyopyknosis and stained brown, and show rounded, crescent, or irregular shape (200×) (B); the periportal endothelial cells (arrow) and hepatocytes are apoptotic in the I/R group (200×) (C), and apoptotic cells are detected around the central vein (arrow) in the I/R group (400×) (D).

The apoptosis rate of sinusoidal endothelial cells in the I/R group was significantly higher than that of the hepatocytes (17.53% ± 1.19% vs. 5.61% ± 0.85%, *P* < 0.001; Fig 11).

### Western blotting

**Fig 12:**
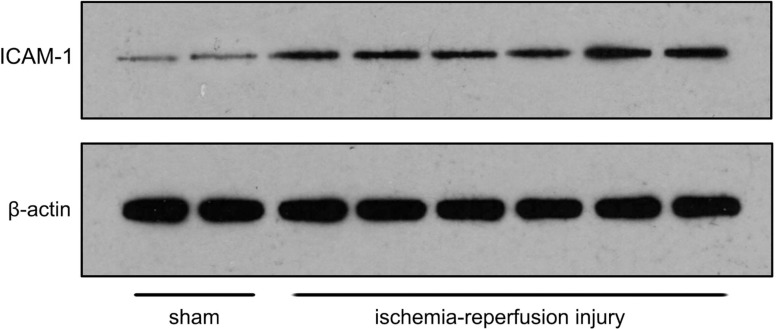
The gray value of ICAM-1 expression in the liver is significantly higher in the I/R group than in the SO group.

The gray value of ICAM-1 expression in the liver was significantly higher in the I/R group than in the SO group (29.60 ± 0.94 vs. 16.90 ± 1.14, *P* < 0.001; Fig 12).

### CEUS results

**Fig 13:**
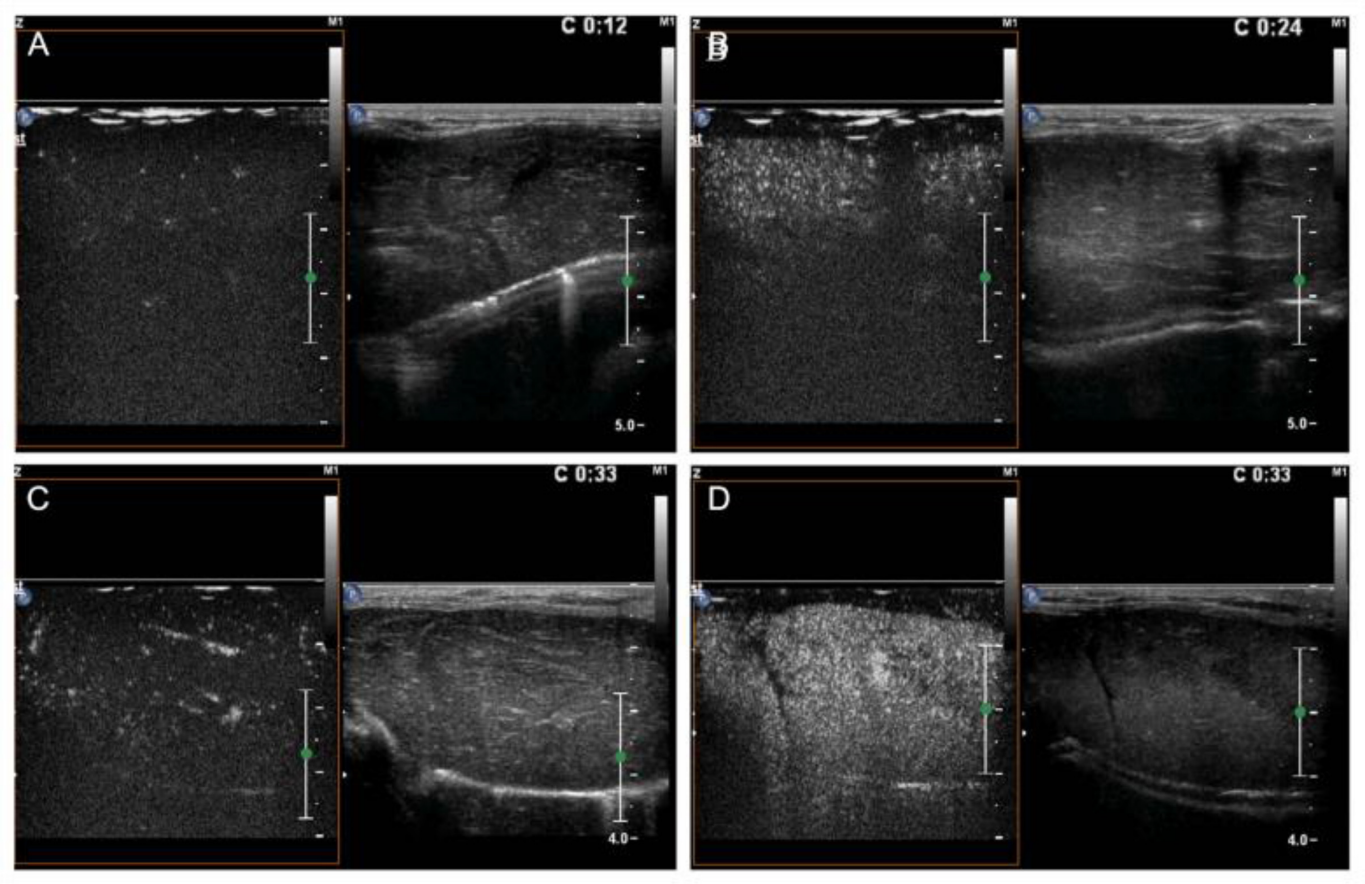
The imaging of CEUS shows the enhancement intensity of the targeted nanobubbles is significantly higher in the I/R group (D) than in the other three groups (A: common nanobubbles enhancement intensity in the SO group, B: targeted nanobubbles enhancement intensity in the SO group, C: common nanobubbles enhancement intensity in the I/R group).

**Fig 14:**
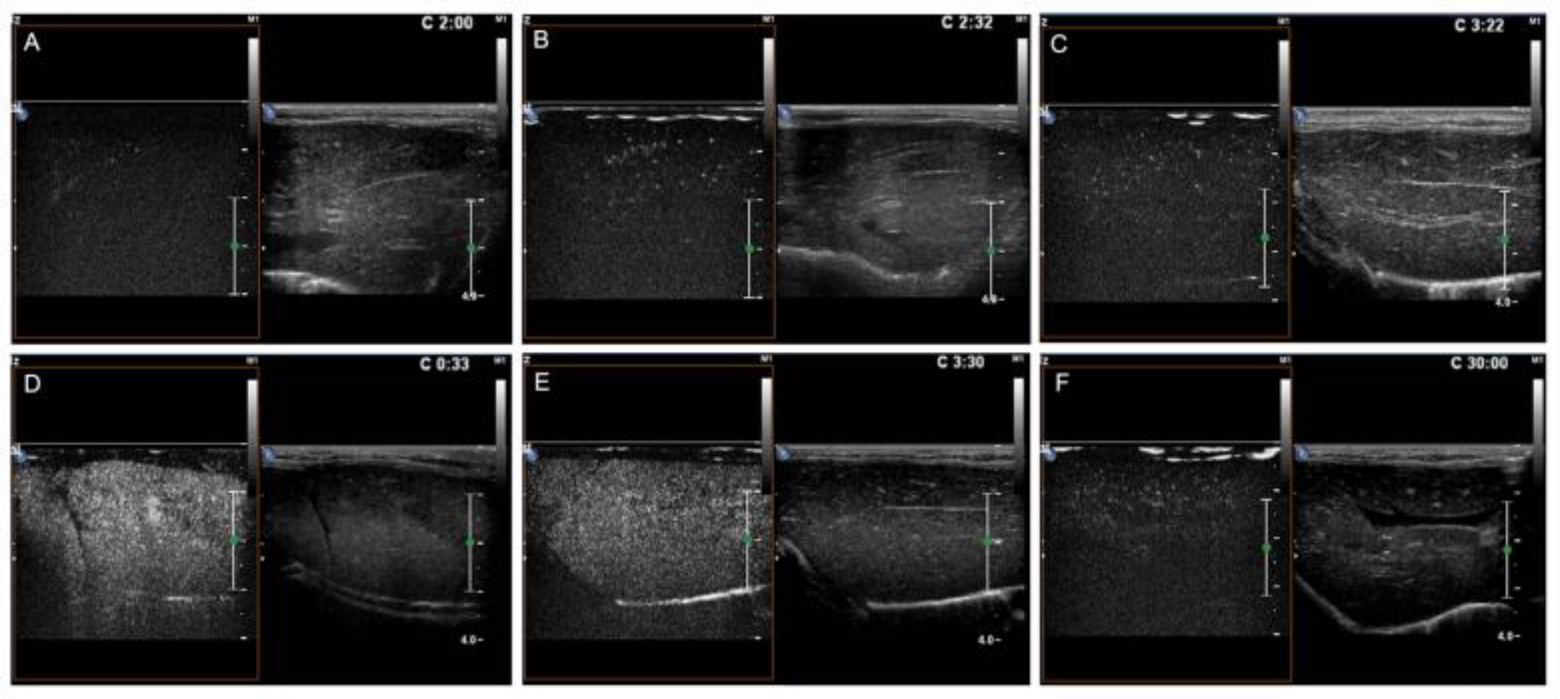
The time of duration is 120 s for the common nanobubbles in the SO group (A), 152 s for the targeted nanobubbles in the SO group (B), and 202 s for the common nanobubbles in the I/R group (C). Echogenicity of the targeted nanobubbles in the I/R group is significantly enhanced with a time to peak of 33 s (D); the enhancement intensity remains undiminished at 210 s (E) and the time of duration is more than 20 mins (22.67 ± 2.52 min). The values are significantly higher than that of the above three groups (F).

The enhancement intensity of the targeted nanobubbles was significantly higher in the I/R group than in the other three groups (Fig 13). The targeted nanobubbles showed a long-time stable aggregation and efficient echogenicity enhancement in the I/R group. The time of enhancement duration was 22.67 ± 2.52 min, which was significantly longer than those of the other groups (Fig 14). Peak intensity, time to peak, and time of duration of the targeted and the common nanobubbles in the SO and I/R groups are shown in Tables 1–3.

**Table 1:** Comparison of the peak intensity (PI) of the nanobubbles (mean ± standard deviation).

	PI (dB)	*F*	*P*
SO common nanobubble	8.64 ± 1.09	91.107	0.000
SO targeted nanobubble	10.10 ± 0.78		
I/R common nanobubble	7.86 ± 0.56		
I/R targeted nanobubble	17.26 ± 1.06^a^^b^		

^a^Compared with I/R common nanobubble, *P*<0.001

^b^Compared with SO targeted nanobubble, *P*<0.001

**Table 2:** Comparison of the time to peak of the nanobubble (mean ± standard deviation).

	Time to peak (s)	*F*	*P*
SO common nanobubble	22.11 ± 0.86	45.593	0.000
SO targeted nanobubble	17.58 ± 1.13		
I/R common nanobubble	24.60 ± 0.54^c^		
I/R targeted nanobubble	20.66 ± 0.84^a^^b^		

^a^Compared with I/R common nanobubble, *P*<0.001

^b^Compared with SO targeted nanobubble, *P*<0.001

^c^Compared with SO common nanobubble, *P*<0.001

**Table 3:** Comparison of the time of duration of the nanobubble (mean ± standard deviation).

	Time of duration (min)	*t*	*P*
I/R common nanobubble	3.13 ± 0.42	13.263	0.005
I/R targeted nanobubble	22.67 ± 2.52		

**Fig 15:**
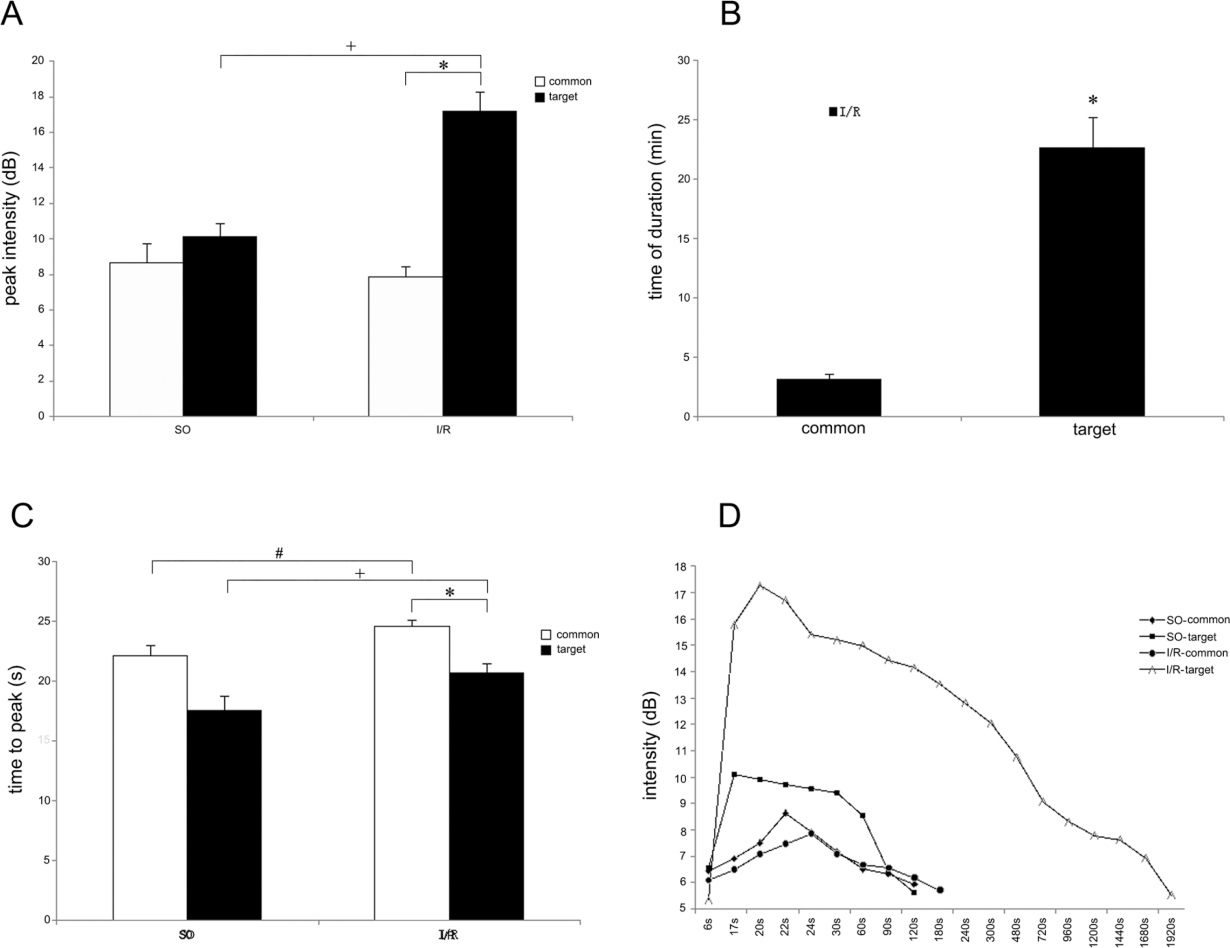
The parameters of liver perfusion were analyzed using time-intensity curve of QLAB software. The imaging of CEUS shows that the ^**※**^peak intensity of the targeted nanobubbles is significantly higher than that of the common ones in the I/R group, *P*<0.001; the ^**+**^peak intensity of the targeted nanobubbles in the I/R group is significantly higher than that in the SO group, *P*<0.001 (A); the ^**※**^time of duration of the targeted nanobubbles is significantly longer than that of the common ones in the I/R group, *P* = 0.005 (B); the ^**+**^time to peak of the targeted nanobubbles is delayed in the I/R group compared to the SO group, *P* = 0.002; the ^**#**^time to peak of the common nanobubbles is delayed in the I/R group compared with the SO group, *P* = 0.010; the ^**※**^time to peak of the targeted nanobubbles is significantly earlier than that of the common ones in the I/R group, *P*<0.001 (C). Comparison of enhancement intensity at different times between the two contrast agents in the I/R and SO groups (D).

The peak intensity and the time of enhancement duration of the targeted nanobubbles were significantly higher than that of common nanobubbles in the I/R group (Fig 15A and 15B). The peak intensity and the time of enhancement duration of the two contrast agents did not differ significantly in the SO group (*P* = 0.241). The time to peak of the targeted the and common nanobubbles were delayed in the I/R group compared with the SO group (*P* = 0.002, *P* = 0.010, Fig 15C). However, the time to peak was significantly earlier in the targeted nanobubbles than in the common nanobubbles in the I/R group (*P* < 0.001). The curve shows that the enhancement intensity and the time of enhancement duration of the targeted nanobubbles were significantly higher in the I/R group than in the other three groups (Fig 15D).

## Discussion

Nanoscale ultrasound contrast agents can spread into the extravascular space. However, due to the poor acoustic reflectivity, nanoscale bubbles are only detectable by ultrasound waves at a high dose in the lesion area. The nanobubbles prepared in our study employ phospholipids as the shell membrane. The thin membrane has a good property of “compression-expansion” under ultrasound. It is filled with the octafluoropropane gas for the production of effective scattering signals. The key technology to prepare targeted nanobubbles is to connect the targeting ligand to its surface. There are three connections: electrostatic adsorption, covalent adsorption, and avidin-biotin connection. This study used the avidin-biotin method [28] to stably bind the targeting ligand (anti-ICAM-1 antibody) to the surface of phospholipid contrast agents, without changing its biological activity and physiological characteristics. Due to a strong affinity between biotin and avidin, whose affinity is known for four independent biotin binding sites, it can combine with more nanobubbles, amplifying the signals, and thus improving the sensitivity of detection of ultrasound contrast agents. More than 95% of contrast agents prepared in our study has a particle size within 200 nm, and effectively accumulate in the lesions and enhance long enough.

Inflammation is characterized by the activation, migration, and infiltration of leukocytes. The aggregation and infiltration of leukocytes are mediated by the interaction between the leukocyte adhesion molecules and the vascular endothelial cell receptors. Microbubbles targeting the inflammatory markers, such as E-selectin/P-selectin and ICAM-1/VCAM-1, have been used in the quantitative evaluation of inflammation of kidney, heart, and colon [29–32]. E-selectin and P-selectin are involved in the regulation of release, recruitment, and rolling of leukocytes in vascular walls during inflammation. ICAM-1 and VCAM-1 play a critical role in the leukocyte migration to tissues by capturing the rolling and migrating leukocytes. Our nanobubbles were designed based on the leukocyte behaviors in the late stage of inflammation.

The hypoxia/reoxygenation experiment of HHSECs *in vitro* is to simulate the cold preservation of liver transplantation. Immunofluorescence staining demonstrated that ICAM-1 is expressed in the HHSECs and the extracellular matrix after hypoxia/reoxygenation injury at a low temperature. In our study, ICAM-1 was a specific antigen in the inflammation of endothelial cells, and a confocal microscope showed that the targeted nanobubbles specifically bond to the damaged surfaces of liver sinusoidal endothelial cells, displaying its good targeting ability.

Our study also confirmed that microcirculation dysfunction could occur in the early stage of liver IRI. The liver sinusoidal blood flow decreased or stopped and the expression of ICAM-1 was upregulated. This is due to the hypoxia after IRI, thrombosis, and oxygen radicals generated, which stimulated the neutrophils, monocytes, and endothelial cells to produce platelet-activating factor, endothelin, and cytokines [tumor necrosis factor, interleukin (IL)-1, IL-6, IL-8], thus contributing to the upregulation of ICAM-1 in liver sinusoidal endothelial cells, sinus small vein endothelial cells, and hepatocytes. The binding of ICAM-1 and the ligands LFA-1 and Mac-1 on the surface of neutrophil promotes the rolling, adhesion, and emigration of neutrophils, which results in inflammatory injuries and aggravates the damage of the liver sinusoidal endothelial cells and hepatocytes [33].

In our study, the targeted nanobubbles can recognize and specifically bind to the lesions at the molecular level, producing specific enhanced image in the target area, thus to improve the detection sensitivity and specificity of ultrasound on the lesions. During the CEUS procedure, targeted nanobubbles not only can pass through the blood circulation smoothly and steadily, selectively accumulate in target lesions and cells, and increase imaging effect, but also can stably accumulate in the target lesions for a long time and effectively enhance. In view of this, the targeted nanobubbles are better for a detailed ultrasound examination of the lesion, can perform early differential diagnosis to the normal and damaged livers at the molecular level, and can improve the sensitivity and specificity of diagnostic ultrasound greatly.

## Conclusion

We successfully prepared a nanoscale ultrasound contrast agent containing anti-ICAM-1 antibody. This targeted nanobubble is stable and shows good binding ability to the HHSECs *in vitro*. Animal experiments the feasibility of early detection of liver blood reperfusion injury in rabbits using the targeted CEUS technique.
